# Comparison of COVID-19 Severity in Vaccinated and Unvaccinated Patients during the Delta and Omicron Wave of the Pandemic in a Romanian Tertiary Infectious Diseases Hospital

**DOI:** 10.3390/healthcare11030373

**Published:** 2023-01-28

**Authors:** Violeta Briciu, Adriana Topan, Mihai Calin, Roxana Dobrota, Daniel-Corneliu Leucuta, Mihaela Lupse

**Affiliations:** 1 Department of Infectious Diseases, “Iuliu Hatieganu” University of Medicine and Pharmacy, 400348 Cluj-Napoca, Romania; 2 The Clinical Hospital of Infectious Diseases, 400348 Cluj-Napoca, Romania; 3 Department of Medical Informatics and Biostatistics, Iuliu Hatieganu University of Medicine and Pharmacy, 400349 Cluj-Napoca, Romania

**Keywords:** COVID-19, vaccination, disease severity, mortality, comorbidities

## Abstract

Romania has a poor uptake of COVID-19 vaccination in its population. The study objectives were to evaluate the differences between vaccinated and unvaccinated hospitalized COVID-19 patients with regard to disease severity, intensive care need, and mortality during the fourth and the fifth wave of the pandemic associated with the Delta and Omicron variants of concern. A retrospective study on a cohort of hospitalized COVID-19 patients was performed in a Romanian tertiary hospital for infectious diseases. Multivariate logistic regression models were built predicting severe/critical COVID-19, intensive care need, and death as a function of vaccination status and adjusted for age, comorbidities, and wave of the pandemic. 2235 COVID-19 patients were included, and vaccination status, as a primary vaccination or a booster dose, was described in 750 (33.5%). Unvaccinated patients were older, with more cardiovascular and endocrine diseases, a longer duration of hospitalization, a higher percentage of severe/critical COVID-19, need for intensive care, and death (*p* < 0.05). The multivariate logistic regression models adjusted for age and comorbidities showed higher odds ratio for severe/critical COVID-19, intensive care need, and mortality in unvaccinated versus vaccinated patients. Our results support vaccination to prevent severe outcomes associated with COVID-19 due to both variants of concern.

## 1. Introduction

The COVID-19 pandemic put an unprecedented pressure on health care systems worldwide. Data provided by the Romanian National Institute of Public Health show that 67,237 deaths and 3,291,986 documented COVID-19 cases occurred in Romania between the start of the pandemic and 13 November 2022 [1]. Romania, according to the most recent EU study, spends less on health than any other EU country, has the lowest gross domestic product per capita expenditure on healthcare in EU, has among the highest death rates from preventable and treatable causes [2], and is associated with one of the lowest rates of vaccination coverage among member nations [3]. As the nationwide COVID-19 vaccination program progressed, the difficulties became clear [4]. The Romanian population showed extreme reluctance towards being vaccinated against COVID-19 due to the propagation of false information and a low level of education regarding vaccination prophylaxis. The lack of education was associated with the absence of prophylactic programs for infectious diseases in immunocompromised or immunocompetent adults. Before the beginning of the COVID-19 pandemic, the influenza vaccine was the only vaccine funded by the National Health system for adults within the risk groups [5]. All these aspects might have contributed to the poor levels of COVID-19 vaccination, with Romania holding the second to last position in EU countries with a cumulative uptake of the primary course in the total population as of 21 November 2022 of 42.4% compared to 73.1% in EU/EEA and an uptake of the first booster vaccine dose in the total population of 9.2% compared to 54.5% in EU/EEA [6].

According to the official data from the Romanian National Institute of Public Health, the fourth wave of the pandemic started in Romania at the beginning of September 2021, with a peak in the last week of October [7]. The fourth wave of the pandemic due to the Delta variant of concern (VOC) put great pressure on the health care system in Romania. On the 20 October, Romania reached a peak for the fourth wave of 15,022 daily new confirmed COVID-19 cases (in other words, 785.38 daily new cases per 1,000,000 inhabitants) [8]. After a decrease in daily new cases in December 2021, the fifth wave started in Romania abruptly after the winter holidays, at the beginning of January 2022 [8].

On the 26 November 2021, the World Health Organization (WHO) classified the B.1.1.529 variant as a VOC, due to potential immune escape and a potentially increased transmissibility compared to Delta and assigned it the label Omicron [9]. Starting from the 48th week of 2021, the first cases of infection with Omicron VOC were detected by sequencing in Romania. From the 1st week of 2022, Omicron VOC represented more than 60% of the sequenced samples [10].

The Omicron variant is the most genetically divergent SARS-CoV-2 variant detected in significant numbers during the pandemic to date. The phenotypic impacts of Omicron VOC are increased transmissibility, reduced risk of hospitalization and severe disease, increased risk of reinfection [11]. It has multiple mutations which are known to impact the action of neutralizing antibodies. In vitro studies confirm that the neutralizing capacity of vaccinee (primary series) and convalescent sera against Omicron is significantly reduced relative to previous SARS-CoV-2 VOC [12].

Omicron can partially evade the protective effects of antibodies elicited by vaccination or previous natural infection according to factors such as the number of vaccinations or time since the last vaccination, thus leaving large portions of the population susceptible to infection or reinfection.

Information on the decreased vaccine effectiveness for Omicron, hospitalization and death in previously vaccinated people has led to an even more reduced uptake of the vaccine in Romania, starting from the beginning of 2022 [13]. COVID-19 vaccines used in Romania are the vaccines approved for use in EU: mRNA-based (BNT162b2, mRNA- 1273) and adenovirus vector (ChAdOx1, Ad26.COV2.S).

Increasing awareness through mass media campaigns on the benefit of SARS-CoV-2 vaccination, based on real-life data, represents a potential tool for increasing vaccination acceptance in the general population in Romania. Mass media campaigns can produce positive changes in health-related behaviors across large populations [14].

The main objectives of the present study were to evaluate differences between vaccinated versus unvaccinated hospitalized patients with regard to COVID-19 disease severity, intensive care need and mortality during the fourth and the fifth wave of the pandemic and to evaluate vaccine protection during the fifth (Omicron VOC) wave in hospitalized patients.

## 2. Materials and Methods

### 2.1. Study Design and Setting

A retrospective study on consecutive hospitalized patients was performed in The Clinical Hospital of Infectious Diseases Cluj-Napoca. The Clinical Hospital of Infectious Diseases Cluj-Napoca is an academic tertiary monospecialty hospital that provides medical services for patients with infectious pathology from Cluj County and neighboring counties. Starting from March 2020, the 200-bed hospital (with an ICU unit of 10 beds extended to 20 beds during the pandemic) was transformed by a Health Ministry order into the first-line hospital for COVID-19 patients in Cluj County, dedicated exclusively to COVID-19.

### 2.2. Participants

Inclusion criteria were diagnosis of COVID-19 (based on a positive SARS-CoV-2 molecular diagnostic or rapid antigen test), age ≥ 12 years old (as COVID-19 vaccination started for teenagers ≥ 12 years old on the 1 June 2021 in Romania), and hospitalization between the 1 September 2021–31 May 2022. Exclusion criteria: diagnosis of reinfection SARS-CoV-2.

### 2.3. Variables

Data collected were age, gender, duration of hospitalization, comorbidities (cardiovascular, diabetes mellitus, obesity, pulmonary, renal, hepatic, rheumatological, neurological, cancer, immunosuppression), vaccination status: unvaccinated, incomplete primary vaccination (in other words, one out of two doses for vaccines that have 2 doses in the primary series), complete primary vaccination, and booster.

The severity of COVID-19 was defined as asymptomatic, mild (without pneumonia), medium (with non-severe pneumonia), and severe/critical (severe: tachypnea with >30 breaths/min or oxygen saturation < 93% at rest or PaO_2_/FIO_2_ < 300 mmHg; critical: respiratory failure requiring invasive or non-invasive mechanical ventilation, shock, or other organ failure that requires intensive care), according to the first World Health Organization classification [15] and adopted in Romania by a Health Ministry Order on the management of COVID-19. Disease severity was established at the end of hospitalization. Comorbidities (cardiovascular, diabetes mellitus, endocrine, hepatic, neurological, obesity, pulmonary, renal, rheumatological, cancer and immunosuppression) were classified into groups by the clinical investigators, the infectious diseases specialists, based on each diagnosis found in the electronic file of the patients and using the International Statistical Classification of Diseases and Related Health Problems 10th Revision [16]. The length of stay in the ICU and clinical outcome (unfavorable to death or favorable with discharge) were recorded. The study interval was divided based on national data into fourth wave (1 September 2021–15 January 2022) and fifth wave (16 January 2022–31 May 2022).

The study was approved by the Ethics Committee of the Clinical Hospital of Infectious Diseases Cluj-Napoca.

### 2.4. Statistical Analysis

Categorical data were presented as counts and percentages. Non-normally distributed quantitative data were presented as median and interquartile range. Comparisons between two independent groups concerning categorical data were performed with chi-squared test or Fisher exact tests (in cases where the expected frequencies were below 5 in more than 20% of the expected cells, as well when any expected frequency was less than one). Comparisons between two independent groups concerning non-normally distributed quantitative data were performed with the Wilcoxon rank sum test. Multivariate logistic regression models were built predicting death, severe/critical cases, or intensive care, as a function of vaccination status (unvaccinated/incomplete vaccination, complete vaccination, and booster) and adjusted for age ≥65 years, and comorbidities (cardiovascular, diabetes, obesity, pulmonary, renal, hepatic, rheumatological, neurological, immunosuppression and cancer) and the wave of the pandemic (4th or 5th). Furthermore, multivariate logistic regression models adjusted for age and comorbidities in hospitalized patients during wave 5 was carried out in order to investigate the effectiveness of vaccination on Omicron VOC. For all the models, multicollinearity was checked with variance inflation factors. Each model was presented by odds ratios, with 95% confidence intervals and p-values. A multiple quantile regression model was built predicting hospitalization time as a function of the number of vaccination doses and the pandemic wave, and adjusted for age and comorbidities, similar to the logistic regression models. The bias corrected and accelerated confidence intervals of the quantile regression model were computed by bootstrapping. For all statistical analyses, *p*-values below 0.05 were considered statistically significant. R environment for statistical computing and graphics version 4.1.2. [17] was used for statistical analyses.

## 3. Results

A total of 2235 patients were included in the study group based on inclusion and exclusion criteria: 1355 patients during the fourth pandemic wave and 880 patients during the fifth pandemic wave. Demographic, clinical data, comorbidities and vaccination status are presented in Table 1.

Vaccination, either complete primary vaccination or a booster dose, was described in 750 (33.5%) out of the 2235 patients evaluated in the study group. Unvaccinated patients were older, with more cardiovascular and endocrine diseases, a longer duration of hospitalization, a higher percentage had a severe/critical form of COVID-19, a higher need for intensive care, and a higher percentage of death (*p* < 0.05).

A higher percentage of vaccinated patients had a diagnosis of cancer (*p* < 0.05), while endocrine diseases were more frequent in unvaccinated patients. No difference was found between vaccinated and unvaccinated patients in regard to associated diabetes, obesity, hepatic, neurological, pulmonary, renal and rheumatological diseases.

**Figure 1:**
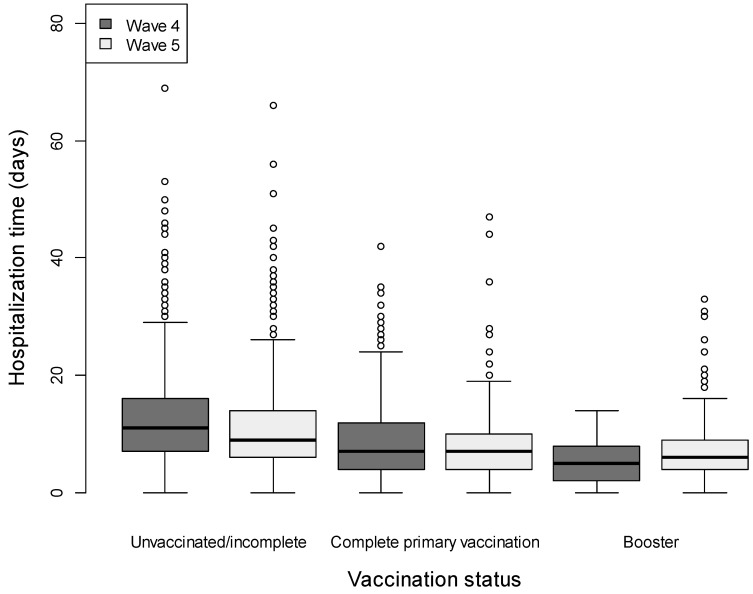
Duration of hospitalization in the three subgroups of patients (unvaccinated/incomplete vaccinated, complete primary vaccination, and booster) during the fourth and the fifth wave of the pandemic. Box represents median observations (horizontal rule) with 25th and 75th percentiles of observed data (top and bottom of box). The length of each whisker represents values up to 1.5 times the interquartile range.

We analyzed the differences in duration of hospitalization between unvaccinated/vaccinated (primary and booster) patients hospitalized during the fourth and the fifth wave, respectively, and the results are presented in Figure 1. In the multiple quantile regression analysis, there was a significantly shorter duration of hospitalization for a higher number of vaccination doses (primary or booster) compared to incomplete/unvaccinated—with a median reduction of 2.33 and 3.44 days in the length of stay, respectively, adjusted for age and comorbidities, as presented in Table 2. Furthermore, there was a significantly shorter duration of hospitalization for the fifth wave compared to the fourth wave (Table 2)—with a median reduction of 1.67 days of hospitalization. 

**Figure 2:**
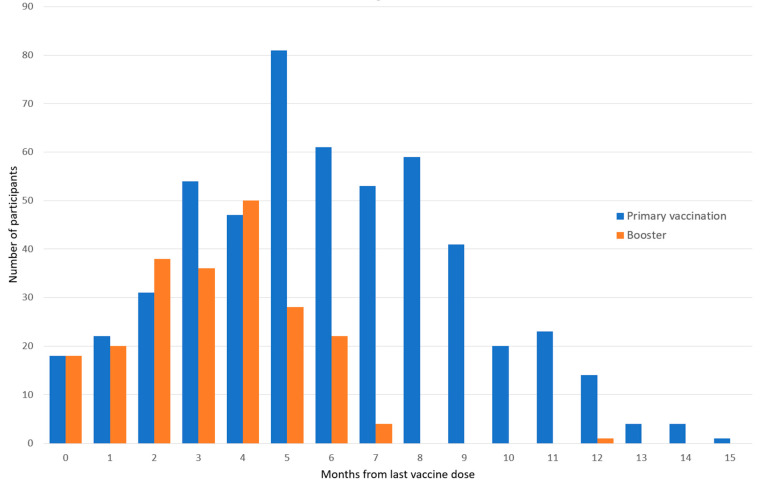
Time interval represented in months between the date of last vaccine administered (primary vaccination or booster vaccination) and hospitalization date.

The interval between the day of the last vaccine shot (primary or booster vaccination) and the date of hospitalization had a median of 184 (IQR 122–257) days and 115 (IQR 75–150) days, respectively, and is represented as intervals in months (Figure 2).

As, apart from vaccination status, patients present different comorbidities as risk factors for a severe outcome, we have adjusted the regression models to patient age and associated diseases in further analyses.

Results of the multivariate logistic regression models predicting severe/critical disease as a function of vaccination status and adjusted for age, comorbidities and pandemic wave is presented in Table 3. The higher odds of reduction in severe/critical disease in vaccinated versus unvaccinated patients remained significant, even after adjustment for numerous confounders.

A significant reduction in intensive care need was found in vaccinated versus unvaccinated patients (Table 1, *p* < 0.001) in the univariate analysis. The results of the multivariate logistic regression models predicting intensive care need as a function of vaccination status and adjusted for age, comorbidities and wave are presented in Table 4. The higher odds of reduction in intensive care need in vaccinated versus unvaccinated patients, remained significant, even after adjustment for numerous confounders.

A significant reduction in mortality was found in vaccinated versus unvaccinated patients (Table 1, *p* < 0.001) in the univariate analysis. The results of the multivariate logistic regression models predicting death as a function of vaccination status and adjusted for age, comorbidities, and wave are presented in Table 5. The higher odds of reduction in mortality in vaccinated versus unvaccinated patients remained significant, even after adjustment for numerous confounders.

### Pandemic Wave 5 Analysis (Omicron VOC)

The results of the multivariate logistic regression models predicting severe/critical disease as a function of vaccination status and adjusted for age and comorbidities in patients hospitalized during the fifth wave are presented in Table 6. The higher odds of reduction in severe/critical disease in vaccinated versus unvaccinated patients also remained significant, even after adjustment for all specified confounders, within the fifth wave.

The results of the multivariate logistic regression models predicting intensive care need as a function of vaccination status and adjusted for age and comorbidities in patients hospitalized during the fifth wave of the pandemic is presented in Table 7. The higher odds of reduction in intensive care need in vaccinated versus unvaccinated patients also remained significant, even after adjustment for all specified confounders, within the fifth wave.

The results of the multivariate logistic regression models predicting death as a function of vaccination status and adjusted for age and comorbidities in patients hospitalized during the fifth pandemic wave is presented in Table 8. The higher odds of reduction in mortality in vaccinated versus unvaccinated patients also remained significant, even after adjustment for all specified confounders, within the fifth wave.

## 4. Discussion

Vaccination continues to be the most reliable way of avoiding severe disease and reducing mortality from COVID-19. By the beginning of September 2022, more than two-thirds of the world population had received at least one dose of a COVID-19 vaccine [18]. Besides the COVID-19 vaccines already in use from the end of 2020, an impressive number of vaccine candidates are in preclinical (198) and clinical (171) development, using different platforms for production [19]. In spite of evidence of the benefits of vaccination, the Romanian population was very reluctant towards being vaccinated. The impact at the population level and on the health care system was most evident during the Delta VOC predominance, as, at the end of October 2021, Romania held the first position globally in terms of daily new COVID-19 deaths per million persons (7-day rolling average of 22.9/million people on the 29 October 2021) [8]. The WHO sent experts to Romania to evaluate the ongoing situation, including the status of the COVID-19 vaccination campaign, and possible explanations for this dramatic reality were addressed [20]. In spite of medical information from clinical studies and real life, nothing could defeat the spread of misinformation that increased the reluctance towards being vaccinated [21]. A study performed in Romania in January 2022 tried to identify the determining factors behind the refusal of vaccination, offering a sociological analysis [22]. Apart from conspiracy theories and fake news, in clinical practice one of the most evoked reason for non-vaccination decision encountered was that vaccinated people also get the disease, need hospitalization, and may even die due to COVID-19. During the Omicron VOC predominance, another reason arguing the non-vaccination decision was that Omicron infection is a mild disease. As a consequence, an important decrease in daily COVID-19 vaccine doses at the national level (from 30,000 in January 2022 to below 10,000 doses of vaccine administered/day) was reported, starting from mid-February 2022, corresponding to the predominance of Omicron VOC in Romania [23].

The main objective of the present study was to bring real-life data from a Romanian hospital in an attempt to inform the impact of vaccination status on COVID-19 severity and outcome in hospitalized patients, and with the hope of increasing vaccination acceptance, based on clinical data.

Data published by UK Health Security Agency at the end of 2021 showed that, after three doses of vaccine, the risk of hospitalization for a symptomatic patient identified with Omicron was reduced by 68% compared to similar individuals with Omicron who were not vaccinated [24]. Though the risk of hospitalization for vaccinated patients persisted, it was reduced compared to unvaccinated patients.

Vaccination, either complete primary vaccination or a booster dose, was described in 750 (33.5%) out of the 2235 patients evaluated in our study group. We found statistically significant older patients in the unvaccinated group (Table 1). COVID-19 vaccination in adults aged 60 years and above in Romania reached 46.8% at the end of November 2022 in comparison to vaccination in Iceland and Ireland, which reached 100% in the same age group [25].

More endocrine diseases were present in the group of unvaccinated patients (Table 1, *p* < 0.001). Patients with an endocrine disease mainly presented Hashimoto’s thyroiditis. Even before the COVID-19 pandemic, the known association between infection and autoimmune diseases has stimulated a debate as to whether autoimmune diseases might also be triggered by vaccines [26]. The potential mechanisms through which COVID-19 vaccine triggers autoimmunity include molecular mimicry, the production of particular autoantibodies and the role of certain vaccine adjuvants [27]. A study on 354 individuals showed no significant increase in the prevalence of anti-nuclear antibodies, anticardiolipin antibodies or and anti-beta-2 glycoprotein I antibodies, or autoimmune diseases in subjects who were vaccinated, 7–9 months after complete immunization [28]. Previous studies have revealed that SARS-CoV-2 infection could trigger autoimmunity [29]. A systematic review published recently identified 52 previously vaccinated patients diagnosed with subacute thyroiditis [30], but whether the association between the COVID-19 vaccine and autoimmune phenomena is coincidental or causal remains to be elucidated. In this context, many patients with preexisting autoimmune thyroiditis refused vaccination in Romania as a consequence of controversial information.

As regards patients with cancer, we found a significant difference regarding increased percentage in the vaccinated group (Table 1, *p* = 0.039). That difference might be explained by a possibly higher percentage of vaccinated patients in the population with cancer compared to the general population. Patients with cancer were prioritized for vaccination and were more compliant with vaccination, as a higher risk of severe evolution was described in this group [31,32]. Our multivariate logistic regression model predicting severe/critical disease, intensive care need, and death in cancer patients showed an OR adjusted of 1.5, 2.2, and 1.71, respectively. We did not perform a separate analysis on subgroups of patients with solid/hematological malignancies, type of tumor, therapy, and present status of malignant disease (remission, curative setting, advanced active disease or palliation). Preda et al. (2022) published data on patients with cancer also hospitalized in our unit at the beginning of the pandemic and confirmed a more severe evolution for COVID-19 in cancer patients in three multivariate analyses performed, with factors associated with impaired overall survival like ECOG performance status and radiotherapy [33].

The percentage of severe/critical disease was higher in unvaccinated patients compared to vaccinated ones. We used the first World Health Organization classification for COVID-19 severity. The most severe form of disease for each patient during hospitalization was recorded by the study investigators. For example, a patient hospitalized in a moderate state, which evolved to a critical state during hospitalization, and then discharged as asymptomatic, was recorded as a critical state. There are markers proposed to identify patients at risk of developing severe/critical COVID-19 that could help a clinician, such as the alveolar–arterial oxygen gradient, that was found to be more appropriate than PaO_2_/FiO_2_ [34].

Duration of hospitalization is an important indicator of disease severity and of the impact on health care services. A longer hospitalization is associated with higher costs, increased hospital and ICU bed occupancy rate (with unavailability of hospital beds for other patients that need medical assistance), and risk for healthcare-associated infections. Until the end of 2021, there have been 4,689,809 COVID-19-related hospital days and 474,713 COVID-19-related ICU days in Romania, with an estimated total cost of hospitalization EUR 1,358,088,411, a tremendous pressure on the healthcare system, but also on the national economy and society [35]. Our data show that unvaccinated patients had a longer in-hospital stay. Each day of hospitalization that could be avoided represents a benefit both for the patient, as well as for the healthcare system through the reduction of hospitalization costs. Patients who have received a primary vaccination spent less time in hospital (a 2.33-day reduction in the median duration of hospitalization), and patients who received a booster vaccination spent even less time in hospital (a median reduction of 3.44 days, which clinically is extremely important). Wave 5, with Omicron VOC predominance, was associated with a shorter duration of hospitalization as compared to wave 4, in both unvaccinated and vaccinated patients.

The booster dose vaccination campaign started in Romania at the beginning of October 2021, with extreme reluctance in the population due to the impact in social media of news regarding infection and death in previously vaccinated people. Infection in vaccinated patients was first described in the efficacy studies both on primary vaccination [36] and booster vaccination [37]. Thomas et al., showed that vaccine efficacy decreased with increasing time after the second dose [38]. A multitude of published studies evaluated the effectiveness of different vaccines in real life, during the circulation of different VOC and in different populations [39,40,41,42]. Vaccine effectiveness against laboratory-confirmed COVID-19 is lower for Omicron than for Delta, but during both waves, vaccine effectiveness was significantly lower among patients who received their second vaccine dose ≥180 days compared with those vaccinated more recently. Furthermore, vaccine effectiveness increased following a third dose and was highly effective during both Delta- and Omicron-predominant periods at preventing COVID-19–associated hospitalizations (94% and 90%, respectively) [43].

Data published by the UK Health Agency, February 2022, presented vaccine effectiveness against mortality with Omicron VOC estimated in vaccinated (all vaccines combined) compared to unvaccinated individuals (50 years and older): at 25+ weeks following the second dose, vaccine effectiveness was around 60% while, at 2 or more weeks following a booster, vaccine effectiveness was 95% against mortality [44].

Questions remain regarding waning immunity and the duration of immunity after COVID-19 vaccination and/or disease. Increased risk of reinfection was associated with Omicron VOC [11]. Published data from UK showed a higher risk of reinfections in the Omicron dominant period with an estimated rate of reinfections (per 100,000 participants days at risk) of 180.3 compared to 11.7 in the pre-Omicron period [45]. However, virus neutralization by sera from people who have experienced a combination of infection and complete vaccination (primary series), or vaccinated people who have received boosters, remains at least partially effective in neutralizing Omicron in vitro [46,47].

In order to exclude from the analyses the bias of increasing immunity by natural infection in association to vaccination, we have excluded from the study group patients with laboratory confirmed previous COVID-19. Of course, undiagnosed mild infections in patients’ history cannot be ruled out.

The multivariate logistic regression models adjusted for age and comorbidities showed higher OR for severe/critical COVID-19, need for intensive care and mortality in unvaccinated versus vaccinated study group patients (primary vaccination and booster dose). The OR is higher when comparing unvaccinated patients to booster dose than to primary vaccination, data reinforcing the benefit of boosting for preventing severe outcomes. Though data was collected, we did not perform a separate analysis on vaccine type.

Apart from vaccination, we found in the multivariate logistic regression models significant statistical values for a severe form of COVID-19 in patients older than 65 years, with diabetes, obesity, cardiovascular, neurological, and malignant diseases and in infections acquired in wave 4 as compared to wave 5 (Table 3). Reduced risk of hospitalization and severe disease in Omicron infection was published by WHO in the epidemiological update on February 2022 [11], based on available data from South Africa [48] and England [49]. Although diabetes could be an independent risk factor for COVID-19 severity [50], patients with diabetes often have other comorbidities such as obesity and cardiovascular diseases that could add to the increased risk of severe COVID-19 [51]. The evidence reported in an umbrella review of systematic reviews suggested that cardiovascular diseases and certain cardiovascular risk factors including hypertension, diabetes mellitus, renal disease, liver disease, cerebrovascular disease, obesity, smoking history and current smoking are associated with a higher likelihood of severe COVID-19 and/or mortality with COVID-19. [52]. Similar results were obtained on the multivariate logistic regression models performed during the 5th pandemic wave: apart from vaccination status, significant statistical values for severe COVID-19 were found in patients older than 65 years (OR 5 (3.27–7.74), with cardiovascular and neurological diseases (Table 6).

Regarding intensive care need, we included in the analyses patients that were actually transferred in the ICU. On the other hand, the 10- transformed to 20-bed capacity of the ICU was seldom overwhelmed, especially during wave 4, and patients with criteria for ICU were managed in the infectious disease department in collaboration with intensivists. That could bring a limitation in interpretation of the analyses on intensive care needs. ICU bed capacity can be rapidly and dramatically increased in a pandemic crisis and the only option is to transiently transform non-ICU beds into ICU beds [53].

Apart from vaccination status, the following risk factors were associated with higher mortality in our study group multivariate analyses: age older than 65 (OR 4.37 (95% CI 2.93–6.67), obesity, cardiovascular, neurological, and malignant diseases, infections acquired in wave 4 as compared to wave 5. Omicron infection was independently associated with a lower risk for in-hospital mortality, but older patients were more affected by Omicron (OR adjusted for death 14.74).

Although on the 1 September 2022 European Medicine Agency authorized across the EU the first two adapted bivalent Original/Omicron BA.1 vaccines [54], the WHO recommended in mid-October 2022 that countries should not delay implementing the first or second boosters while waiting for access to variant-containing vaccines. There is a greater benefit in ensuring that persons at high risk of developing severe COVID-19 receive a booster 4–6 months after the previous dose rather than extending this interval in anticipation of a variant-containing vaccine.

### Limitations and Strengths

The retrospective observational nature of our study precludes any causation claim and implies the possibility of residual confounding. Nevertheless, the multivariate models that were employed adjusted for numerous comorbidities and the imbalances between the vaccinated and unvaccinated groups (e.g., age ≥ 65 years, cardiovascular disease were more frequent in the nonvaccinated group, and cancer was more frequent in the vaccinated group in the univariate analysis). The multivariate models cannot correct for all differences between the groups. Due to overwhelmed ICUs, some patients that might have needed intensive care and were managed in the infectious disease department were not counted as such in our analyses.

Nevertheless, our study strengths are represented by the adjustment for a large number of confounders and by its large size. As far as we know, this is the first study that brings real-life data from Romania on the impact of vaccination status on the severity level of COVID-19, intensive care need, and evolution in hospitalized patients during the Delta and Omicron VOC waves. Moreover, our data show that vaccinated patients had a shorter in-hospital stay. Each day of hospitalization that could be avoided represents a benefit, both for the patient, as well as for the healthcare system and society with the reduced burden of hospitalization costs. Based on the results of our real-life study performed in a first line COVID-19 hospital, we hope to increase awareness on the benefit of SARS-CoV-2 vaccination, both in the medical community not involved in the management of severe COVID-19 patients, as well as in the general population. Increasing awareness and developing educational programs on the benefit of vaccination strategies in respiratory infectious diseases with pandemic potential represent a major goal for the healthcare systems worldwide. Investigators intend to involve in public health campaigns to raise awareness and understanding about COVID-19 health issues, and mobilize support for action in local community.

## 5. Conclusions

COVID-19 vaccination conferred protection against the severe/critical form of the disease, intensive care need, and death due to both the Delta and Omicron VOC (wave four and five of the pandemic in Romania) and was associated with a reduced duration of hospitalization. Until adapted vaccines are largely available for new virus variants, our results support vaccination with available vaccines during Omicron variant domination to prevent severe outcomes associated with COVID-19. Healthcare workers and public health departments may use present scientific evidence for prevention strategies that improve outcome for COVID-19 patients and reduce the burden of healthcare costs.

## Figures and Tables

**Table 1:** Patients’ demographic, clinical data, comorbidities and vaccination status.

	All Patients			Wave 4			Wave 5		
Characteristics	Not Vaccinated (n = 1485)	Vaccinated (n = 750)	*p*-Value	Not Vaccinated (n = 1036)	Vaccinated (n = 319)	*p*-Value	Not Vaccinated (n = 449)	Vaccinated (n = 431)	*p*-Value
Age (years), median (IQR)	68 (52–79)	65 (48–75)	<0.001	65 (51–75)	63 (50.5–73)	0.046	74 (60–83)	67 (44.5–76.5)	<0.001
Age ≥ 65 years, n (%)	851 (57.31)	385 (51.33)	0.007	532 (51.35)	148 (46.39)	0.122	319 (71.05)	237 (54.99)	<0.001
Sex (male), n (%)	628 (42.29)	336 (44.8)	0.258	457 (44.11)	151 (47.34)	0.311	171 (38.08)	185 (42.92)	0.144
Vaccine doses, n (%)			<0.001			<0.001			<0.001
	0/1 *: 1485 (100)	0/1: 0 (0)		0/1: 1036	0/1: 0 (0)		0/1: 449 (100)	0/1: 0 (0)	
	2 **: 0 (0)	2: 533 (71.07)		2: 0	2: 277 (86.83)		2: 0 (0)	2: 256 (59.4)	
	3 ***: 0 (0)	3: 217 (28.93)		3: 0	3: 42 (13.17)		3: 0 (0)	3: 175 (40.6)	
Comorbidities									
Cardiovascular, n (%)	975 (65.66)	458 (61.07)	0.033	676 (65.25)	207 (64.89)	0.906	299 (66.59)	251 (58.24)	0.01
Diabetes, n (%)	341 (22.96)	171 (22.8)	0.931	245 (23.65)	82 (25.71)	0.453	96 (21.38)	89 (20.65)	0.79
Endocrine, n (%)	126 (8.48)	35 (4.67)	<0.001	98 (9.46)	17 (5.33)	0.021	28 (6.24)	18 (4.18)	0.17
Hepatic, n (%)	99 (6.67)	46 (6.13)	0.629	66 (6.37)	16 (5.02)	0.375	33 (7.35)	30 (6.96)	0.823
Cancer, n (%)	128 (8.62)	85 (11.33)	0.039	69 (6.66)	35 (10.97)	0.011	59 (13.14)	50 (11.6)	0.488
Neurological, n (%)	238 (16.03)	113 (15.07)	0.556	144 (13.9)	44 (13.79)	0.962	94 (20.94)	69 (16.01)	0.06
Obesity, n (%)	472 (31.78)	220 (29.33)	0.237	377 (36.39)	115 (36.05)	0.912	95 (21.16)	105 (24.36)	0.257
Pulmonary, n (%)	217 (14.61)	107 (14.27)	0.826	130 (12.55)	50 (15.67)	0.15	87 (19.38)	57 (13.23)	0.014
Renal, n (%)	100 (6.73)	58 (7.73)	0.384	66 (6.37)	27 (8.46)	0.196	34 (7.57)	31 (7.19)	0.829
Rheumatological, n (%)	55 (3.7)	40 (5.33)	0.071	37 (3.57)	21 (6.58)	0.02	18 (4.01)	19 (4.41)	0.768
Hospitalization time (days), median (IQR)	10 (6–16)	6 (4–10)	<0.001	11 (7–16)	7 (4–11)	<0.001	9 (6–14)	6 (4–9)	<0.001
Disease severity, n (%)			<0.001			<0.001			<0.001
Asymptomatic	3 (0.2)	3 (0.4)		1 (0.1)	0 (0)		2 (0.45)	3 (0.7)	
Mild	137 (9.23)	201 (26.8)		68 (6.56)	78 (24.45)		69 (15.37)	123 (28.54)	
Medium	373 (25.12)	299 (39.87)		245 (23.65)	108 (33.86)		128 (28.51)	191 (44.32)	
Severe/critical	972 (65.45)	247 (32.93)		722 (69.69)	133 (41.69)		250 (55.68)	114 (26.45)	
ICU stay, n (%)	252 (16.97)	53 (7.07)	<0.001	166 (16.02)	23 (7.21)	<0.001	86 (19.15)	30 (6.96)	<0.001
Died, n (%)	217 (14.61)	36 (4.8)	<0.001	163 (15.73)	21 (6.58)	<0.001	54 (12.03)	15 (3.48)	<0.001

IQR, interquartile range; * 0/1, 0 means unvaccinated, 1 incomplete vaccination (one dose in vaccination with two doses in primary vaccination); ** 2, complete primary vaccination; *** 3, booster vaccination; ICU, intensive care unit; Wave 4, Delta VOC (variant of concern) wave of pandemic; Wave 5, Omicron VOC wave of pandemic.

**Table 2:** Multiple quantile regression model predicting duration of hospitalization time in function of pandemic wave and vaccination status, adjusted for age and comorbidities.

Predictor	Coefficient (Days)	95% CI	*p*-Value
Age ≥ 65 years	2.22	1.67–3.00	<0.001
Cardiovascular	1.44	0.50–2.00	<0.001
Diabetes	1.00	0.25–2.00	0.001
Obesity	0.33	0.00–1.00	0.315
Pulmonary	−0.22	−1.00–0.33	0.520
Renal	0.33	−0.50–1.67	0.474
Hepatic	0.22	−0.67–2.00	0.657
Rheumatological	1.00	−0.25–2.25	0.236
Neurological	1.88	1.00–2.68	<0.001
Cancer	1.67	1.00–3.00	<0.001
Immunosuppression	2.78	0.00–13.00	0.123
Doses (2 vs. 0/1)	−2.33	−3.00–−2.00	<0.001
Doses (3 vs. 0/1)	−3.44	−4.00–−2.68	<0.001
Wave (5 * vs. 4 #)	−1.67	−2.15–−1.00	<0.001

CI, confidence interval; *, Omicron wave; #, Delta wave; * 0/1, 0 means unvaccinated, 1 incomplete vaccination (one dose in vaccination with two doses for primary vaccination); ** 2, complete primary vaccination; *** 3, booster vaccination; all variables are included in the model.

**Table 3:** Results of the multivariate logistic regression model predicting severe/critical COVID-19.

Characteristics	OR Adjusted	(95% CI)	*p*
Age ≥ 65 years	2.28	(1.81–2.87)	<0.001
Cardiovascular	2.05	(1.64–2.57)	<0.001
Diabetes	1.77	(1.39–2.26)	<0.001
Obesity	1.36	(1.1–1.69)	0.005
Pulmonary	1.3	(0.99–1.72)	0.062
Renal	1.3	(0.88–1.92)	0.19
Hepatic	0.99	(0.68–1.46)	0.964
Rheumatological	1.49	(0.92–2.42)	0.107
Neurological	1.78	(1.34–2.38)	<0.001
Cancer	1.5	(1.08–2.1)	0.016
Immunosuppression	1.72	(0.52–5.52)	0.361
Vaccine doses (0/1 vs. 2)	3.23	(2.56–4.0)	<0.001
Vaccine doses (0/1 vs. 3)	5.55	(8.33–3.85)	<0.001
Pandemic wave (5 * vs. 4 ^#^)	0.44	(0.36–0.54)	<0.001

OR, odds ratio; CI, confidence interval; *, Omicron wave; ^#^, Delta wave; all variables are included in the model.

**Table 4:** Results of the multivariate logistic regression model predicting intensive care need.

Characteristics	OR Adjusted	(95% CI)	*p*
Age ≥ 65 years	1.06	(0.78–1.45)	0.703
Cardiovascular	1.92	(1.39–2.68)	<0.001
Diabetes	1.2	(0.89–1.59)	0.219
Obesity	1.59	(1.2–2.09)	0.001
Pulmonary	1.57	(1.13–2.15)	0.006
Renal	1.27	(0.8–1.97)	0.292
Hepatic	1.24	(0.76–1.95)	0.361
Rheumatological	1.2	(0.62–2.16)	0.569
Neurological	1.79	(1.29–2.46)	<0.001
Cancer	2.2	(1.49–3.2)	<0.001
Immunosuppression	2.77	(0.59–9.7)	0.14
Vaccine Doses (0/1 vs. 2)	2.33	(1.64–3.33)	<0.001
Vaccine Doses (0/1 vs. 3)	5.88	(3.13–14.29)	<0.001
Wave (5 * vs. 4 ^#^)	1.17	(0.89–1.54)	0.253

OR, odds ratio; CI, confidence interval; *, Omicron wave; ^#^, Delta wave; all variables are included in the model.

**Table 5:** Results of the multivariate logistic regression model predicting death.

Characteristics	OR Adjusted	(95% CI)	*p*
Age ≥ 65 years	4.37	(2.93–6.67)	<0.001
Cardiovascular	1.87	(1.26–2.83)	0.002
Diabetes	0.93	(0.67–1.28)	0.665
Obesity	1.49	(1.09–2.03)	0.013
Pulmonary	1.36	(0.95–1.93)	0.087
Renal	1.09	(0.65–1.77)	0.722
Hepatic	1.41	(0.81–2.35)	0.203
Rheumatological	0.94	(0.42–1.88)	0.875
Neurological	2.3	(1.65–3.18)	<0.001
Cancer	1.71	(1.09–2.62)	0.017
Immunosuppression	3.8	(0.5–17.77)	0.13
Vaccine doses (0/1 vs. 2)	2.77	(1.85–4.35)	<0.001
Vaccine doses (0/1 vs. 3)	3.57	(1.78–8.33)	<0.001
Wave (5 * vs. 4 ^#^)	0.51	(0.37–0.69)	<0.001

OR, odds ratio; CI, confidence interval; *, Omicron wave; ^#^, Delta wave; all variables are included in the model.

**Table 6:** Results of the multivariate logistic regression model predicting severe/critical COVID-19 during the fifth pandemic wave.

Characteristics	OR Adjusted	(95% CI)	*p*
Age ≥ 65 years	5	(3.27–7.74)	<0.001
Cardiovascular	1.69	(1.13–2.52)	0.011
Diabetes	1.41	(0.96–2.07)	0.078
Obesity	1.33	(0.9–1.96)	0.151
Pulmonary	1.28	(0.85–1.93)	0.24
Renal	0.83	(0.47–1.49)	0.541
Hepatic	0.82	(0.45–1.49)	0.525
Rheumatological	1.54	(0.71–3.35)	0.273
Neurological	1.73	(1.16–2.6)	0.008
Cancer	1.45	(0.9–2.35)	0.124
Immunosuppression	3.82	(0.8–17)	0.079
Vaccine Doses (0/1 vs. 2)	2.94	(2.04–4.34)	<0.001
Vaccine Doses (0/1 vs. 3)	4	(2.56–6.25)	<0.001

OR, odds ratio; CI, confidence interval; all variables are included in the model.

**Table 7:** Results of the multivariate logistic regression model predicting intensive care need in patients hospitalized during the fifth pandemic wave.

Characteristics	OR Adjusted	(95% CI)	*p*
Age ≥ 65 years	3.18	(1.66–6.45)	<0.001
Cardiovascular	1.3	(0.76–2.29)	0.347
Diabetes	0.94	(0.56–1.55)	0.82
Obesity	1.15	(0.67–1.93)	0.596
Pulmonary	1.61	(0.98–2.62)	0.056
Renal	1	(0.45–2.03)	0.999
Hepatic	1.61	(0.75–3.22)	0.201
Rheumatological	1.6	(0.51–4.17)	0.371
Neurological	1.91	(1.17–3.1)	0.009
Cancer	2.08	(1.18–3.62)	0.01
Immunosuppression	4.96	(0.65–25.12)	0.073
Vaccine Doses (0/1 vs. 2)	1.96	(1.19–3.33)	0.009
Vaccine Doses (0/1 vs. 3)	5.26	(2.5–12.5)	<0.001

OR, odds ratio; CI, confidence interval; all variables are included in the model.

**Table 8:** Results of the multivariate logistic regression model predicting death in patients hospitalized during the fifth pandemic wave.

Characteristics	OR Adjusted	(95% CI)	*p*
Age ≥ 65 years	14.74	(4.05–97.73)	<0.001
Cardiovascular	1.76	(0.87–3.86)	0.133
Diabetes	0.49	(0.22–0.98)	0.055
Obesity	0.95	(0.44–1.92)	0.885
Pulmonary	1.27	(0.67–2.34)	0.447
Renal	0.33	(0.07–1.05)	0.105
Hepatic	1.62	(0.57–3.94)	0.318
Rheumatological	2.29	(0.62–6.77)	0.165
Neurological	2.03	(1.12–3.63)	0.018
Cancer	2.59	(1.32–4.95)	0.005
Immunosuppression	19.08	(0.8–199.62)	0.023
Vaccine Doses (0/1 vs. 2)	2.86	(1.4–6.25)	0.006
Vaccine Doses (0/1 vs. 3)	3.33	(1.47–9.09)	0.008

OR, odds ratio; CI, confidence interval; all variables are included in the model.

## Data Availability

Data availability available on request, due to to restrictions, e.g., privacy or ethical.
